# Effect of sintering temperature on color stability and translucency of various zirconia systems after immersion in coffee solution

**DOI:** 10.1371/journal.pone.0313645

**Published:** 2024-11-14

**Authors:** Rashin Giti, Sina Mosallanezhad

**Affiliations:** 1 Department of Prosthodontics, Faculty of Dentistry, Shiraz University of Medical Sciences, Shiraz, Fars, Iran; 2 Student Research Committee, Faculty of Dentistry, Shiraz University of Medical Sciences, Shiraz, Fars, Iran; University of Vigo, SPAIN

## Abstract

**Background and aim:**

Achieving the aesthetic standards in tooth-colored restorative materials requires close attention to their color, translucency, and resistance to discoloration. This study aimed to evaluate the effect of sintering temperature on color stability and translucency in zirconia systems with low, high, and ultra-high translucencies.

**Methods:**

This experimental study was conducted on 60 zirconia disks with low, high and ultra-high translucencies (n = 20 per group), each group divided into subgroups to be sintered at either 1450°C or 1550°C (n = 10 per subgroup). Baseline color and translucency parameters were measured, the specimens were then immersed in coffee solution for 30 days, and the measurements were repeated post-immersion. Changes in color (ΔE) and translucency (ΔTP) were calculated via CIELAB formula and compared by using two-way ANOVA and Tukey’s post hoc test (α = 0.05).

**Results:**

Results of two-way ANOVA showed that the ΔE was significantly different among the three zirconia translucencies (P<0.001), but no significant difference was found between the two sintering temperatures (P = 0.712). Additionally, the interaction between zirconia type and sintering temperature was not statistically significant for ΔE (P = 0.264). The low-translucency group showed significantly greater ΔE than the high-translucency and ultra-high-translucency groups (P<0.05), while the high- and ultra-high-translucency groups were not significantly different in this regard (P>0.05). Regarding the ΔTP, two-way ANOVA showed that the difference was not statistically significant either among the three zirconia types (P = 0.4430) or between the two sintering temperatures (P = 0.4544). Nor was the interaction between zirconia type and sintering temperature statistically significant (P = 0.5505).

**Conclusion:**

It was concluded that sintering temperature had no effect on color and translucency changes after immersion in coffee. Whereas zirconia type significantly affected the color changes after immersion in coffee; with the higher-translucency zirconia types being significantly more color-stable than the low-translucency zirconia.

## 1. Introduction

In recent years, zirconium oxide or zirconia-based restorations have gained significant popularity owing to their superior strength, fracture resistance, and great biocompatibility [1, 2]. Computer-aided designed/computer-aided manufactured (CAD-CAM) monolithic zirconia crowns and fixed partial dentures serve as excellent alternatives to conventional bilayered core-ceramic systems due to their heightened fracture strength [3, 4], that mitigates issues related to veneering porcelain chipping [3, 5]. However, they may fall short in terms of translucency and aesthetic appeal in comparison [6].

Pure zirconia appears in three crystallographic forms based on temperature: monoclinic (at room temperature up to 1170°C), tetragonal (1170°C to 2370°C), and cubic (2370°C to its melting point). Transition from tetragonal to monoclinic form induces a 3% to 5% volume expansion, resulting in cracks and fractures in the zirconia structure. Consequently, pure zirconia is unsuitable for use in dental crowns. This can be prevented by stabilizing zirconia in cubic or tetragonal forms and inhibiting the transition into monolithic form through the incorporation of various stabilizing oxides such as Yttrium oxide (Y_2_O_3_) [7–10]. This stabilization at room temperature facilitates a phenomenon called transformation toughening, which enhances the fracture toughness of zirconia, making it superior to traditional porcelain materials [11].

Yttria-stabilized tetragonal zirconia polycrystals (Y-TZP), also known as partially stabilized zirconia, contain between 2 and 5 mol% Y_2_O_3_. The term ‘partially’ refers to the addition of Yttria in concentrations lower than those required for complete cubic zirconia stabilization. Generally, increasing the Yttria content in Y-TZP elevates its translucency and optical properties, while decreasing its flexural strength and overall mechanical properties [12–15].

Aesthetics are paramount for clinicians when selecting tooth-colored restorative materials. This includes considerations such as color, translucency, and susceptibility to discoloration from prolonged exposure to staining food and beverages like coffee and tea [16]. Human perception of color appearance is inherently subjective. The color appearance of ceramic primarily depends on the spectral reflectance generated by light scattering at the surface. Translucency stands as a critical optical property in achieving a satisfactory shade match with natural teeth [17]. Light partially scatters or reflects as it passes through an object. The translucency of a material is directly linked to the amount of light that penetrates the object [18]. Translucency is generally evaluated using the translucency parameter (TP) and contrast ratio [19, 20].

The amount of Yttria content is a contributing factor for zirconia systems with different levels of translucency. Additionally, the sintering temperature significantly affects the size and density of zirconia particles, which directly influences the density, porosity, and growth of these particles. Sintering zirconia at higher temperatures can yield larger grain sizes, consequently enhancing translucency and rendering it aesthetically more suitable for monolithic zirconia crowns [21–26]. However, Kim et al. [27] reported that although raising the sintering temperature of zirconia can impact grain size, it may not necessarily improve translucency. The sintering process mainly influences microstructure and flexural strength in monolithic zirconia crowns. Similarly, Cardoso et al. [28] observed that sintering fully stabilized monolithic zirconia at higher temperatures yielded larger grain size without any significant impact on its translucency.

Light scattering is influenced by the relationship between grain size and the wavelength of incident light. As the grain size approaches the wavelength, scattering increases and leads to a reduction in light transmittance. However, when the grain size significantly exceeds the wavelength, scattering becomes inversely proportional to grain size and remains unaffected by the incident light wavelength [29–31].

Low-temperature degradation is considered the primary adverse factor affecting the long-term mechanical properties of zirconia ceramics within the oral cavity. This degradation arises from a gradual transition from the unstable tetragonal phase to the monoclinic phase, which is accelerated by the presence of moisture in the environment [32].

Inokoshi et al. [33] reported that higher sintering temperatures resulted in a larger ZrO_2_ grain size and a greater proportion of cubic crystal structure. It reduced the amount of Yttria content within tetragonal grains, making them more vulnerable to low-temperature degradation. Too et al.’s [34] findings were conflicting about the relationship between sintering temperature and translucency. However, their study revealed that some zirconia systems are more prone to degradation at higher sintering temperatures. A study by Buyukkaplan et al. [35] showed that 28 days of immersion in coffee had no significant effect on translucency of ceramic samples.

The recommended sintering temperature typically ranges between 1400°C and 1600°C, depending on the manufacturer’s guidelines. To improve the translucency of monolithic zirconia crowns, some manufacturers advise raising the sintering temperature. Increasing the sintering temperature and duration results in larger grain sizes, which are more prone to undergo stress-induced transformation into a more stable structure, thereby enhancing the material toughness. Maximum toughness is reached when the grain size is around 1 μm size [9]. If exceeded, the material undergoes spontaneous transformation from the tetragonal to the monoclinic phase, leading to a reduction in stability. Sintering temperature plays a crucial role in grain densification, and thus the mechanical properties of specimen [9, 28]. It may also affect the marginal fit, as ceramics shrink to varying degrees when cooling from high temperature to room temperature [36].

There are controversies in recent findings regarding the effect of sintering temperature on color stability and translucency of different zirconia systems with different translucencies. There is also a gap in research focusing on degradation at room temperature through factors such as extended contact with staining beverages like coffee. Given the absence of studies on the combined effects of zirconia translucency and sintering temperature on color stability and translucency of monolithic zirconia after immersion in coffee solution, the current study was conducted to evaluate the effect of sintering temperature on color stability and translucency in zirconia systems with low, high and ultra-high translucencies after immersion in coffee.

The null hypothesis was that differences in sintering temperature would not affect the color stability and translucency of different zirconia systems with low, high and ultra-high translucencies after immersion in coffee.

## 2. Materials and methods

This experimental study was conducted in March 2024, and data were collected through experiments and measurements. No external data sources were accessed. Ethical approval was obtained from the Ethics Committee of Shiraz University of Medical Sciences (IR.SUMS.DENTAL.REC.1402.037).

### 2.1. Fabrication of specimens

Disk-shaped specimens (2×10 mm) were computer-aided designed (3shape, Copenhagen, Denmark) and milled (CAD-CAM machine, Cori Tec 340i; imes-icor GmbH, Eiterfeld, Germany) out of three types of Y-TZP monolithic zirconia blocks: low-translucency (DD Bio ZW iso, High Strength Zirconia, Dental Direkt, Germany) (Group L), high-translucency (DD Bio ZX^2^ 98, High Translucent Zirconia, Dental Direkt, Germany) (Group H) and ultra-high translucency (DD cubeX^2^, Super High Translucent Zirconia, Dental Direkt, Germany) (Group UH) (Table 1).

**Table 1:** Characteristics of tested zirconia brands.

Brand	Translucency	Code	Manufacturer	Composition
**DD Bio ZW**	Low (opaque)	L	Dental Direct GmbH	ZrO₂ + HfO₂ + Y₂O₃ ≥ 99 Y₂O₃ < 6 Al₂O₃ < 0.5 Other oxides < 1.0
**DD Bio ZX^2^**	High	H	Dental Direct GmbH	ZrO₂ + HfO₂ + Y₂O₃ ≥ 99 Y₂O₃ < 6 Al_2_O_3_ ≤ 0.15 Other oxides < 1.0
**DD cubeX^2^**	Ultra-high	UH	Dental Direct GmbH	ZrO₂ + HfO₂ + Y₂O₃ ≥ 99 Y₂O₃ ≤ 10 Al₂O₃ ≤ 0.01 Other oxides < 1.0

**Fig 1:**
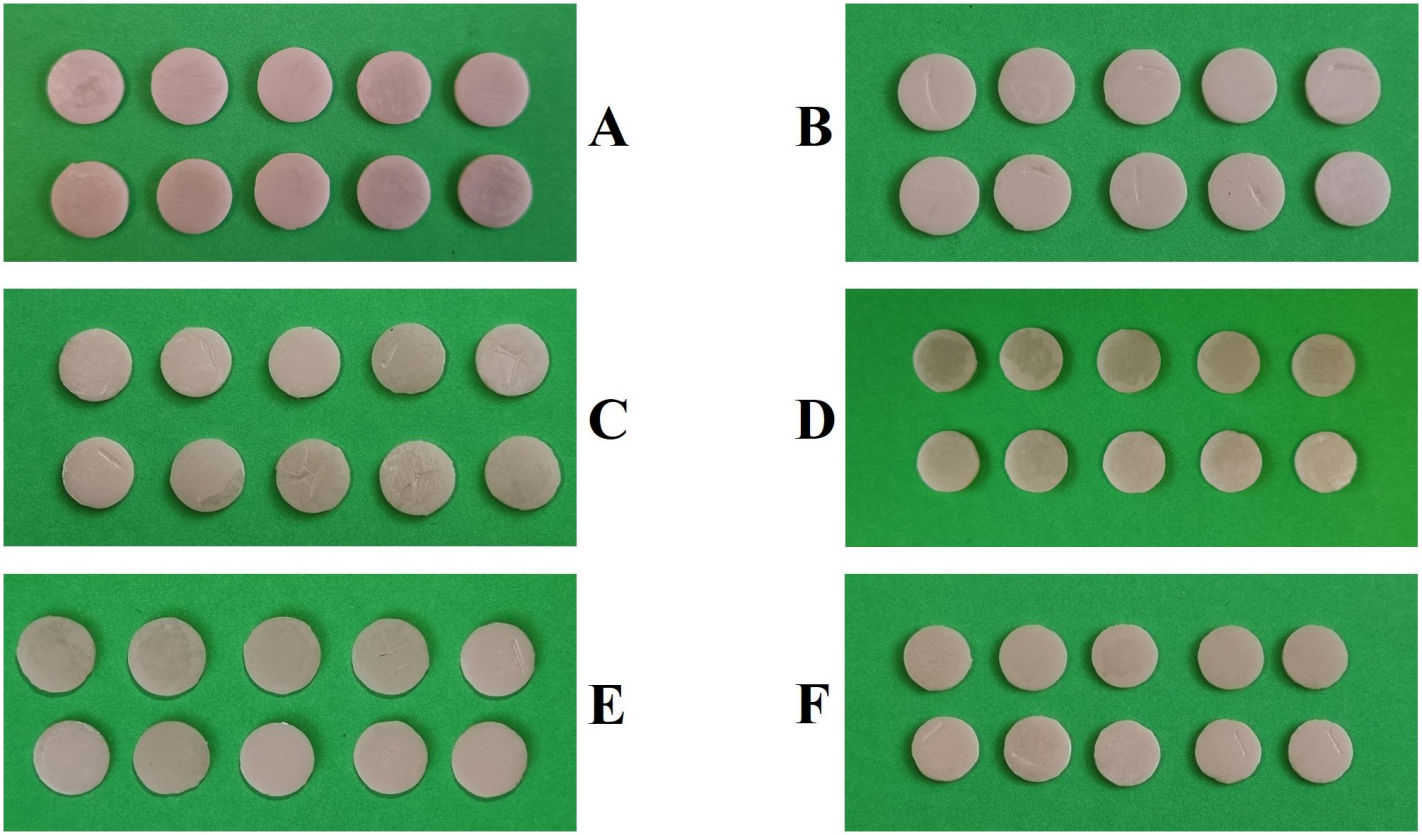
Monolithic zirconia samples in each study group before immersion in coffee: A) Group L, 1450°C, B) group L, 1550°C, C) group HL, 1450°C, D) group H, 1550°C, E) group UH, 1450°C, and F) group UH, 1550°C.

A total of 60 disks, 20 from each zirconia group, were prepared and divided through simple randomization into 2 subgroups (n = 10) to be sintered at either 1450°C or 1550°C (Fig 1). The selection of two sintering temperatures was based on the previous studies [9, 28]. The sintering protocol was started at room temperature, with an 8°C per minute heating rate up to the maximum temperature (1450°C or 1550°C). After a 2-hour step time, the temperature was decreased to room temperature at an 8°C per minute cooling rate in a sintering furnace (Sinterofen HT-S Speed, mihm-vogt, Germany).

To establish a uniform baseline color for each sample, the A2 shade was selected from the VITA shade guide (Vita ZahnFabrik, Bad Säckingen, Waldshut, Germany). As instructed by the manufacturer, the specimens were evenly polished on both sides. They were ultrasonically cleaned in distilled water for 15 minutes and individually air-dried for 15 seconds prior to testing [9, 28].

### 2.2. Color measurement

Baseline color values (L*, a*, b*) were determined against a white (w) and a black (b) background by using a calibrated reflectance spectrophotometer (VITA Easyshade V^®^, Bad Säckingen, Waldshut, Germany) incorporating a D65 illuminant and a 2-degree standard observer to generate the CIELab values. Each specimen underwent three measurements, and the average value was calculated from these three readings. Both color and translucency assessments were conducted simultaneously in a dark room at 24°C by a skilled technician blind to the study groups [37].

The color appearance can be measured scientifically using the Commission Internationale de l’Eclairage (CIE) system [38], where L* is the luminosity axis, a* is the green-red axis (-a = green and +a = red), and b* is the blue-yellow axis (-b = blue and +b = yellow). The TP values were obtained by calculating the color difference between black and white backgrounds via the following equation [39]:

$$\text{TP}={\left[{\left({\text{L}}_{\text{B}}-{\text{L}}_{\text{W}}\right)}^{2}+{\left({\text{a}}_{\text{B}}-{\text{a}}_{\text{W}}\right)}^{2}+{\left({\text{b}}_{\text{B}}-{\text{b}}_{\text{W}}\right)}^{2}\right]}^{1/2}$$ (1)

To ensure consistency, the device was positioned perpendicular to each specimen, and the same side of each disk was measured for all readings. All measurements were done by the same operator. Before each measurement each specimen was washed with distilled water and dried with a paper towel.

The specimens were, then, immersed in coffee solution for 30 days (equivalent to 27 months of daily coffee consumption [40]), which was replaced daily. The coffee solution was freshly prepared each day by dissolving 1.8 g of NESCAFÉ instant coffee powder into 200 ml of 85 ºC water and steering the solution. Following the 30-day period, the specimens were rinsed in distilled water 10 times, wiped with paper towel, and left to dry.

The TP values were remeasured as before and ΔTP was calculated using the following formula:

ΔTP=post−immersionTP–pre-immersionTP (2)

Color measurements were repeated as before, and changes in color values (L, a, b) were calculated. The difference in color appearance for each sample represented the calculus of total color change and was calculated using the following formula [41]:

$$\Delta \text{E}={\left[{\left(\Delta {\text{L}}^{*}\right)}^{2}+{\left(\Delta {\text{a}}^{*}\right)}^{2}+{\left(\Delta {\text{b}}^{*}\right)}^{2}\right]}^{1/2}$$ (3)

Perceptibility of color difference is defined by ΔE values, where 0.5<ΔE<1 is imperceptible, ΔE = 1 is perceptible, and ΔE≤3.7 is the clinically acceptable threshold for a visible color difference [42].

### 2.3. Statistical analysis

Data analysis was conducted by using SPSS software (version 16; SPSS Inc., Chicago, Illinois, USA). Data were presented as mean and standard deviations (mean ± SD) for ΔE and ΔTP. Normal distribution and equality of variances were assessed using the Shapiro-Wilk and Levene’s test, respectively. Two-way ANOVA and Tukey’s post hoc test were used for inter-group comparisons. P values <0.05 were considered to be statistically significant [28].

## 3. Results

**Fig 2:**
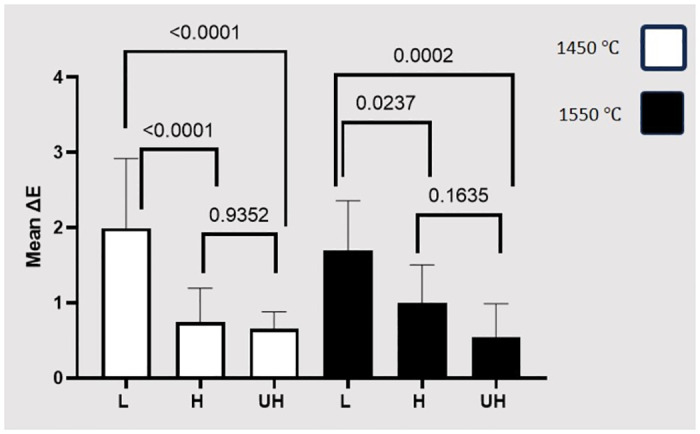
Comparing the means and standard deviations of ΔE with respect to the sintering temperature.

Normal distribution and homogeneity of variances were confirmed by Shapiro-Wilk and Levene’s tests, respectively. The means and standard deviations of the ΔE and ΔTP in each group are presented in Table 2. Two-way ANOVA revealed that ΔE was significantly different among the three zirconia types (P<0.001), while there was no significant difference between the two sintering temperatures (P = 0.712). The interaction between the zirconia type and sintering temperature had no statistically significant effect on ΔE (P = 0.264) (Table 3). Fig 2 compares the ΔE among the three zirconia types in each of the sintering temperatures.

**Table 2:** The means and standard deviations (mean ± SD) of ΔE and ΔTP.

Zirconia type	n	Sintering Temperature	ΔE	ΔTP
BioZW (group L)	10	1450°C	1.99 ± 0.92^a^*	-0.10 ± 1.01 ^c^
	10	1550°C	1.69 ± 0.66^a^	-0.44 ± 0.31 ^c^
ZX2 (group H)	10	1450°C	0.74 ± 0.45^b^	-0.14 ± 0.51 ^c^
	10	1550°C	1.0 ± 0.50^b^	-0.13 ± 0.50 ^c^
CubeX2 (group UH)	10	1450°C	0.65 ± 0.22^b^	-0.04 ± 0.50 ^c^
	10	1550°C	0.54 ± 0.44^b^	-0.04 ± 0.37 ^c^

*Different superscript letters indicate statistical difference in a column.

**Table 3:** The results of two-way ANOVA for comparing the effects of zirconia type and sintering temperature in ΔE parameters.

Variable	Type III sum of squares	df	Mean Square	F	P value
Zirconia type	17.56	2	8.78	27.43	<0.001
Sintering Temperature	0.044	1	0.044	0.138	0.7117
Zirconia type × sintering temperature	0.873	2	0.436	1.36	0.2640

Among the 1450°C sintering temperature subgroup, ΔE of the L group was significantly different from that of the H (P<0.001) and UH groups (P<0.001). However, there was no statistically significant difference between the H and UH groups in this regard (P = 0.9352).

Among the subgroups sintered at 1550°C, the ΔE of the L group was significantly different from those of the H (P = 0.0237) and UH groups (P = 0.0002), while the difference between the H and UH groups was not statistically significant (P = 0.1635).

**Fig 3:**
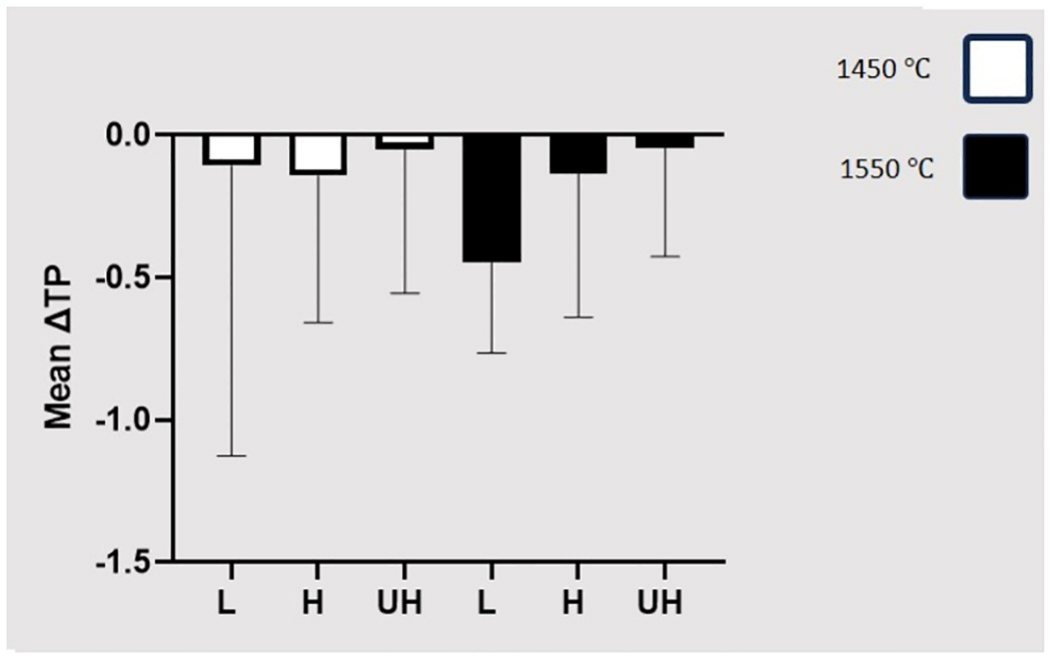
Comparing the means and standard deviations of ΔTP with respect to the sintering temperature.

Regarding the ΔTP, two-way ANOVA showed no significant difference among the three zirconia types (P = 0.4430) or the two sintering temperatures (P = 0.4544). Nor was any significant interaction detected between the zirconia type and sintering temperature (P = 0.5505) (Table 4 and Fig 3).

**Table 4:** The results of two-way ANOVA for comparing the effects of zirconia type and sintering temperature in ΔTP.

Variable	Type III Sum of Squares	df	Mean Square	F	P value
Zirconia	0.558	2	0.279	0.826	0.4430
Sintering temperature	0.192	1	0.192	0.567	0.4544
Zirconia type × sintering temperature	0.407	2	0.204	0.603	0.5505

## 4. Discussion

The null hypothesis was partially rejected as color change was found to be dependent on the zirconia type, but not on the sintering temperature. Furthermore, the changes in translucency were not influenced by either the sintering temperature or zirconia type.

In the present study, the zirconia disks were 2 mm thick. According to Tabatabaian et al. [43], a thickness of 1.6 mm achieves ideal masking ability in vitro. Sasany et al. [44] found that 0.7-mm-thick samples exhibited less translucency in all zirconia groups; while, 1.5-mm-thick specimens showed no significant change in translucency.

Research indicates that the sintering process influences the transparency of monolithic zirconia [5, 21, 45]. Nevertheless, the current study was among the firsts to examine the effects of sintering temperature and zirconia type on color stability and translucency following exposure to a staining beverage.

The most and least color changes were in the low-translucency and ultra-high translucency zirconia types. Yet, they remained below the clinically acceptable threshold in all the study groups (ΔE<3.7) [42]. Regarding the translucency parameter, immersion in coffee decreased the ΔTP in all groups, with the least notable decrease observed in the ultra-high translucency zirconia. However, the differences among the groups were not statistically significant.

According to some previous research, 30 days of immersion in coffee simulates the effect of 2.5 years of coffee consumption, considering the time coffee remains in the oral cavity when sipping [40, 46]. Routine oral hygiene practices are assumed to effectively eliminate or diminish surface discolorations. Therefore, the color and translucency changes observed in this study might be less significant in vivo.

Several studies have assessed the influence of staining beverages on color and translucency of monolithic zirconia [47–50]. Alnassar et al.’s study [49] showed that monolithic zirconia specimens were significantly more discolored by coffee, rather than protein shake, chlorhexidine mouthwash, and a soft drink. It is not only the tannin and chlorogenic acids content in coffee that induces discoloration, but also its low pH level, which further exacerbates the discoloration process [51].

In line with the present study, Al-Zordk et al. [48] observed that coffee thermocycling influenced the color and translucency of different zirconia systems; although the color change remained below the clinically acceptable limits. Additionally, the type of zirconia systems significantly affected the color and translucency change, with 5Y-TZP being less affected than 3Y-TZP. The present study supported these findings, although the effect of zirconia type on translucency was deemed insignificant. This discrepancy can possibly be due to differences in the materials and methods used in the two studies.

On the other hand, Buyukkaplan et al. [35] observed that 28 days of immersion in coffee had no effect on zirconia translucency, possibly due to the difference in the zirconia brands and translucencies.

Low-temperature degradation is the main factor compromising the long-term mechanical properties of zirconia ceramics in the oral cavity. It results from a gradual transition from the unstable tetragonal phase to the monoclinic phase, accelerated by atmospheric moisture [32]. Denry et al. [5] found that increasing the sintering temperature can increase zirconia translucency by enlarging the grain sizes. However, this also makes the material more susceptible to low-temperature degradation. Meanwhile, Stawarczyk et al. [21] detected that increasing the sintering temperature and contrast ratio were directly related, which indicates increasing the sintering temperature would result in reduced translucency.

Variations in chemical structures, grain size, shape, crystalline phase distribution, porosity, and thickness, all impact zirconia’s optical properties, thus causing the differences in ΔE and ΔTP among different zirconia systems [52–54]. Consistent with the current study, several studies have documented that higher Yttria content in zirconia can reduce the surface low-temperature degradation and potentially decrease roughness and infiltration of staining beverages [48, 55, 56]. This can lead to lower color change in high-Yttria zirconia.

In the present study, the low-translucency specimens were 3Y-TZP, and the high-translucency samples were 3Y-TZP with reduced alumina content and La_2_O_3_ dopants. Reduced alumina content prevents the formation of secondary phases of distinct alumina particles in the crystalline structure, thereby, increasing translucency. However, this reduction significantly decreases hydrothermal stability and makes the material more prone to discoloration and loss of translucency over time. The addition of La_2_O_3_ compensates for this issue by promoting the segregation of La^3+^ and Al^3+^ cations at the grain boundaries, consequently enhancing the material’s resistance to hydrothermal degradation. The ultra-high translucency samples were 5Y-TZP, which have significantly higher translucency and greater resistance to hydrothermal aging than 3Y-TZP, due to their higher Yttria content [39, 57–59].

The present study evaluated color differences using the CIELAB formula, where L* represents lightness and corresponds to Munsell’s value, while a* and b* represent chromaticity coordinates. In this context, a* indicates the color position along the red/purple-green/blue axis, and b* indicates the color position along the blue/purple-yellow axis [60]. Currently, the CIEDE2000 color difference formula is recommended as it more accurately reflects color differences perceived by the human eye compared to the CIELAB formula. Furthermore, it provides better adjustments for determining color disparities by addressing inconsistencies in the CIELAB formula [61].

Among the limitations of this study is its in-vitro methodology, which did not simulate the behavior of restorations in the oral environment influenced by saliva. This also precluded the examination of oral hygiene routines, such as tooth brushing, which could affect the color stability. Another limitation could be the use of CIELAB formula to quantify color disparities, rather than the more advanced CIEDE2000 formula, which might have affected the accuracy of findings. Moreover, analysing only a single zirconia brand might restrict the generalization of the obtained results to alternative monolithic zirconia brands. Further research are advised to evaluate the influence of staining beverages on the color stability and translucency of various brands of monolithic zirconia in intraoral settings and using the advanced CIEDE2000 color difference formula.

## 5. Conclusion

Within the limitations of this study and based on the current findings, it can be concluded that sintering temperature does not affect the color change (ΔE) and translucency change (ΔTP) of monolithic zirconia restorations after immersion in coffee solution. However, zirconia type significantly affects color changes after immersion in coffee, with the higher translucency zirconia types being significantly more color stable than the low translucency type. Moreover, the type of zirconia does not affect the translucency change (ΔTP) after immersion in coffee.

## Supporting information

S1 Table Raw data for all study groups. (XLSX)
